# Renaissance of Conventional First-Line Antibiotics in* Salmonella enterica* Clinical Isolates: Assessment of MICs for Therapeutic Antimicrobials in Enteric Fever Cases from Nepal

**DOI:** 10.1155/2017/2868143

**Published:** 2017-09-05

**Authors:** Puspa Raj Khanal, Deepa Satyal, Anjeela Bhetwal, Anjila Maharjan, Shreena Shakya, Snehika Tandukar, Narayan Prasad Parajuli

**Affiliations:** ^1^Department of Laboratory Medicine, Manmohan Memorial Institute of Health Sciences, Kathmandu, Nepal; ^2^Department of Clinical Laboratory Services, Manmohan Memorial Medical College and Teaching Hospital, Kathmandu, Nepal

## Abstract

Enteric fever caused by* Salmonella enterica* is a life-threatening systemic illness of gastrointestinal tract especially in tropical countries. Antimicrobial therapy is generally indicated but resistance towards commonly used antibiotics has limited their therapeutic usefulness. Therefore, we aimed to determine the antimicrobial susceptibility pattern by minimum inhibitory concentration method of common therapeutic regimens against* Salmonella enterica* from enteric fever clinical cases.* Salmonella enterica* clinical isolates recovered from the patients with suspected enteric fever whose blood samples were submitted to microbiology laboratory of Manmohan Memorial Community Hospital, Kathmandu, from March 2016 to August 2016, were studied. These isolates were subjected to antimicrobial susceptibility testing against common therapeutic antimicrobials by Kirby-Bauer disk diffusion method. The minimum inhibitory concentration of ciprofloxacin, azithromycin, chloramphenicol, and cefixime was determined by Agar dilution method based on the latest CLSI protocol. A total of 88 isolates of* Salmonella enterica* were recovered from blood samples of enteric fever cases. Out of them, 74 (84.09%) were* Salmonella* Typhi and 14 (15.91%) were* Salmonella* Paratyphi A. On Kirby-Bauer disk diffusion antimicrobial susceptibility testing, entire isolates were susceptible to cotrimoxazole, cefixime, ceftriaxone, azithromycin, and chloramphenicol. Sixty-four (72.7%)* Salmonella enterica* isolates were nalidixic acid resistant and nonsusceptible to ciprofloxacin and levofloxacin. On MIC determination, four* Salmonella* isolates were ciprofloxacin resistant with MIC 1 *µ*g/ml and two isolates were ciprofloxacin intermediate with MIC 0.5 *µ*g/ml. The MIC range of azithromycin was from 0.125 *µ*g/ml to 2.0 *µ*g/ml, whereas that for chloramphenicol was 2.0 *µ*g/ml–8.0 *µ*g/ml and for cefixime was 0.0075–0.5 *µ*g/ml, respectively. Despite global surge of antimicrobial resistance among* Salmonella enterica* clinical isolates, the level of drug resistance in our study was not so high. However, higher level of NARST strains limits therapeutic use of fluoroquinolones and necessitates the routine monitoring of such resistance determinants in order to effectively and rationally manage enteric fever cases.

## 1. General Background

Enteric fever is a severe systemic and febrile illness, caused by* Salmonella enterica* serovars Typhi and Paratyphi [1]. It remains an important public health problem in the developing countries with inadequate sanitation and safe drinking water [2]. In 2010, 11.9 million typhoid fever illnesses and 129,000 deaths in low and middle income countries were reported due to enteric fever [3].

Antimicrobial agents have long been used to treat the enteric fever and also to reduce mortality associated with severe cases [4]. Traditional antimicrobial regimens previously used for the treatment of enteric fever were chloramphenicol, ampicillin, and trimethoprim-sulfamethoxazole. However the emergence of multidrug resistant (MDR)* Salmonella* in late 1980s restricted the traditional therapeutic options and fluoroquinolones, especially ciprofloxacin, became the alternative option for treating* Salmonella* infections [5]. Unfortunately, the high and indiscriminate use of fluoroquinolones accompanied the evolution of ciprofloxacin resistant strains within their short span of use [3]. Moreover, recently the significant decrease of ciprofloxacin susceptibility has been reported from number of countries, raising a threat of the untreatable enteric fever [6–9].

In Nepal, there is continuous change in the epidemiology of enteric fever during the last few decades [10]. Series of enteric fever outbreaks linked to variable drug susceptibilities have been reported since the first report of MDR* Salmonella* in 1991 [11]. Cephalosporins and macrolides are nowadays the mainstay of therapeutic regimens for enteric fever in Nepal [12]. The major problem of enteric fever treatment in Nepal is the growing rate of fluoroquinolone resistance in* Salmonella* infections [10, 13]. However, notable reports indicating reemergence of the susceptibility towards conventional first-line antimicrobials have raised a new hope in antimicrobial armamentarium [11, 12]. This trend of downsurge of MDR* Salmonella* strains and the revival of conventional first-line drugs provide an opportunity to reevaluate the first-line traditional drugs as therapeutic alternatives in the present scenario. Therefore, this study was intended to determine the spectrum of* Salmonella enterica* serovars among febrile enteric fever patients and their minimum inhibitory concentrations towards commonly used therapeutic antimicrobials at a community based hospital of Kathmandu, Nepal.

## 2. Materials and Methods

A cross-sectional study was conducted at the bacteriology laboratory of department of microbiology, Manmohan Memorial Community Hospital (MMCH), Kathmandu, Nepal. The ethical approval was received from Institutional Review Committee of Manmohan Memorial Institute of Health Sciences before conducting the research. The* Salmonella enterica* clinical isolates recovered from the blood sample of patients with suspected enteric fever during the period of six months (March to August 2016) were included in this study. However* Salmonella enterica* isolated from sample other than blood and duplicate isolates from the same patient were excluded.

### 2.1. Laboratory Methods

The blood samples from suspected enteric fever cases were collected aseptically and cultured in brain heart infusion broth (HiMedia, India) by conventional blood culture technique as described by American Society for Microbiology [ASM] [14]. Isolation and identification of* Salmonella* isolates was performed by standard microbiological techniques including colonial morphology, gram's staining, biotyping, and serotyping (Denka Seiken Co. Ltd., Japan).

### 2.2. Antimicrobial Susceptibility Testing

The antimicrobial susceptibility tests were performed by modified Kirby-Bauer disk diffusion method on Muller Hinton Agar (HiMedia, India) as per CLSI recommendations [15]. The antibiotics tested in this study include ampicillin (10 *µ*g), cefixime (5 *µ*g), ceftriaxone (30 *µ*g), nalidixic acid (30 *µ*g), ciprofloxacin (5 *µ*g), levofloxacin (5 *µ*g), chloramphenicol (30 *µ*g), cotrimoxazole (25 *µ*g), azithromycin (15 *µ*g), and tetracycline (30 *µ*g), respectively. All the antibiotics used were purchased from HiMedia Laboratories, Mumbai, India. Interpretation of antibiotic susceptibility results was made according to standard interpretative zone diameters suggested in CLSI guidelines [15].

### 2.3. Determination of Minimum Inhibitory Concentrations

The minimum inhibitory concentration of respective antimicrobials including ciprofloxacin, chloramphenicol, azithromycin, and cefixime was determined by Agar dilution method as suggested by Andrews [16] based on CLSI guidelines [15]. The antibiotics used were primary reference standard for Nepal and were obtained from Department of Drug Administration (DDA), Government of Nepal, Kathmandu. The tested concentration of the antimicrobials ranged between 0.0075 *µ*g/ml and 32 *µ*g/ml for respective antibiotics. The* Escherichia coli* ATCC-25922 was used in every set of experiment as the part of quality control for susceptibility.

### 2.4. Data Analysis

Data regarding* Salmonella* serotypes, drug susceptibilities and minimum inhibitory concentrations were entered in statistical software. The data were analyzed by using SPSS version 20.0 and interpreted according to frequency distribution and percentage.

## 3. Results

### 3.1. Bacterial Isolates

During the study period, a total of 88* Salmonella enterica* isolates were recovered from blood samples of patients with suspected enteric fever, in which 74 (84.09%) were* Salmonella* Typhi and the remaining 14 (15.91%) were* Salmonella* Paratyphi.

### 3.2. Antibiogram of* Salmonella*

On antimicrobial susceptibility testing by disk diffusion, entire isolates of* Salmonella* Typhi were susceptible to azithromycin, chloramphenicol, cotrimoxazole, cefixime, and ceftriaxone. Similarly entire isolates of* Salmonella* Paratyphi were susceptible to ampicillin, azithromycin, chloramphenicol, cotrimoxazole, tetracycline, cefixime, and ceftriaxone. Among 88 isolates, 72.7% were nalidixic acid resistant* Salmonella* (NARS) which include* Salmonella* Typhi (58, 78.4%) and* Salmonella* Paratyphi (6, 42.9%). The NARS isolates showed reduced susceptibility to fluoroquinolones. Only 21.6% of* Salmonella* Typhi and 57.1% of* Salmonella* Paratyphi isolates were fluoroquinolone susceptible. None of the* Salmonella* isolates were multidrug resistant (MDR) (Table 1).

### 3.3. Minimum Inhibitory Concentrations of* Salmonella*

On MIC determination, entire 88* Salmonella* isolates showed susceptibility towards azithromycin, cefixime, and chloramphenicol. However, four* Salmonella* Typhi were ciprofloxacin resistant with MIC 1 *µ*g/ml and two were intermediate with MIC 0.5 *µ*g/ml. The MIC of azithromycin and cefixime was distributed within 0.125–2 *µ*g/ml and 0.0075–0.5 *µ*g/ml, respectively. Similarly for chloramphenicol, MIC ranged between 2.00 and 8.00 *µ*g/ml. The detailed information regarding the MIC distribution is illustrated in Table 2.

### 3.4. Indicators of* Salmonella* Isolates with Nalidixic Acid and Ciprofloxacin Susceptibility

Among 88* Salmonella* isolates, 27.3% of the isolates that were nalidixic acid and ciprofloxacin susceptible by disk diffusion were also susceptible to ciprofloxacin on MIC determination. However, 65.9% of NARS isolates having ciprofloxacin intermediate on disk diffusion were susceptible on MIC (Table 3).

## 4. Discussion

Kathmandu, the capital city of Nepal, still remains as an endemic region of enteric fever affecting the large population, residents as well as the travelers visiting this area. Enteric fever is the disease of public health concern worldwide and several studies emphasizing the significant burden of enteric fever in developing countries are reported [17]. The variation in the serotypes and susceptibility pattern of* Salmonella* strains along with changing epidemiology has highlighted the importance of susceptibility testing for proper empirical therapy to treat the enteric fever cases.

This study was conducted among the* Salmonella enterica* isolates recovered from the patients suspected of having enteric fever, expecting the findings would have important application in clinical laboratories and medicine in our setting. A total of 88* Salmonella enterica* isolates were recovered, with predominance of* Salmonella* Typhi.* Salmonella* Typhi was found to be the major causative agent of enteric fever in other similar types of studies but with lower distribution rate [12, 18]. However,* Salmonella* Paratyphi strains have also been reported as predominant causes of enteric fever by Pokharel et al. (Paratyphi A 53%) previously from nearby hospital [19]. The serovar variation might be due to the geographical difference, seasonal variation, and study population.

Our study reveals the reemergence of first-line traditional antityphoidal antibiotics against* Salmonella enterica*. Though the drugs were initially susceptible to* Salmonella*, simultaneous resistance was reported from number of countries limiting the therapeutic use of chloramphenicol, cotrimoxazole, and ampicillin [5]. Dave et al. also suggested the significant resistance to chloramphenicol (26% resistant, MIC > 256 mg/l) in their study [20]. However, we observed entire isolates to be susceptible to chloramphenicol (MIC 2–8 *µ*g/ml), cotrimoxazole (100%), and ampicillin (94.6%). Similar result of susceptibility of* Salmonella* strains to chloramphenicol was observed in the study of Neopane et al. [21]. In addition, Badiyal et al. also focused on the reemergence of susceptibility of chloramphenicol including ampicillin and cotrimoxazole in India [22]. In our study, the susceptibility of these drugs was higher and in greater proportion when compared to other similar studies conducted in this region [19, 23]. The higher susceptibility might be due to discontinuation in use of the traditional antibiotics for longer period of time, the loss of high molecular weight self-transmissible plasmid, or the emergence of de novo susceptible strains [22]. This notably depicts the fact of* Salmonella* rolling back to sensitivity against the traditional drugs like chloramphenicol although by limiting the use of routine therapeutic option of fluoroquinolones.

Beside the increased susceptibility of traditional drugs against* Salmonella*, they showed reduced susceptibility to most commonly used therapeutic antimicrobial agents: fluoroquinolones. Out of 88 strains, 6.8%* Salmonella enterica* isolates showed the reduced susceptibility to ciprofloxacin MIC (≥0.5 *µ*g/ml). The rate of nalidixic acid resistance was observed as high (72.7%) but somewhat similar to the result demonstrated by Acharya et al. (80%) and Shrestha et al. (83.1%) [11, 24]. NARS strains have reduced fluoroquinolone susceptibility and nalidixic acid resistance has been used as an indirect indicator of increased minimum inhibitory concentration for ciprofloxacin [25]. Even Acharya et al. from Nepal demonstrated the decrease in ciprofloxacin susceptibility (MICs 0.125–0.5 mg/L) among the NARS strains [24], whilst this study strongly nullifies the use of nalidixic acid as surrogate marker of ciprofloxacin susceptibility. The NARS strains showing reduced susceptibility to ciprofloxacin on disk diffusion were all sensitive on MIC determination of ciprofloxacin (MICs ≤ 0.06 *µ*g/ml). In the study by Chand et al., too, all the nalidixic acid resistant* S.* Typhi isolates were sensitive to ciprofloxacin on disk diffusion [12]. Previously, Accou-Demartin et al. and Hakanen et al. also demoralized the use of the nalidixic acid as screening marker for ciprofloxacin in view of the fact that some resistance characters are not detected by the nalidixic acid disk screening [26, 27]. Recently, perfloxacin disk is recommended as the screening tool of ciprofloxacin susceptibility by CLSI [15], but independent reports regarding its utility from this region are not available.

On the other hand, there are limited studies regarding the efficacy of azithromycin for treating enteric fever, as well as any correlation of MICs with clinical improvement or failure [28, 29]. In Nepal, there is no reported evidence of treatment failure on azithromycin treatment despite the fact that the increase in the MIC of azithromycin was reported from other countries [30]. Concerning all this, we could conclude that there was no resistance and increase in MICs of azithromycin in our context, since the azithromycin MICs was 0.125–2 *µ*g/ml.

Fortunately, none of the* Salmonella enterica* isolates was multidrug resistant (MDR) in this study. Multidrug resistant* Salmonella enterica* isolates (resistant to chloramphenicol, ampicillin, and cotrimoxazole) have been increasingly reported from Asian countries [31, 32]. In Nepal, too, there have been several enteric fever epidemics with changing antibiotic resistance patterns [19, 33]. In the last decade, fluoroquinolones have been the first choice for the treatment of typhoid fever in endemic areas [34]. Subsequently, during the last few years, nalidixic acid resistant strains associated with reduced susceptibility to fluoroquinolones in the patients treated with quinolones have been increasingly reported elsewhere [35] including Nepal [36, 37]. Cephalosporins and macrolides are nowadays the therapeutic choices for enteric fever cases in our region [12], but the emergence of multidrug resistant and extended spectrum *β*-lactamase (ESBL) producing strains has created a therapeutic challenge [19]. Recently, in a multinational phylogenetic analysis of* S*. Typhi clinical strains, H58 clone was found to be responsible for the broader antimicrobial resistance in South and Southeast Asian region and Africa providing the common ancestor of this MDR strain disseminating globally [38].

## 5. Limitations of the Study

The study was conducted with small number of isolates from a single hospital and the sensitivity result of limited number of bacterial isolates might be insufficient for suggesting the antimicrobial therapy for all cases. In addition, all agents subjected for antimicrobial susceptibility testing were not included in MIC testing. The clinical isolates were collected from the suspected patients as their standard healthcare; however, they were not followed up for the treatment outcome. Furthermore, phage typing and genotypic analysis could not be performed for isolates exhibiting resistant characters due to the lack of resources and equipment in our hospital.

## 6. Conclusions

The conventional antityphoidal drugs, third-generation cephalosporins and azithromycin, can be used as an effective empirical therapy for treating enteric fever cases in our setting. The disk diffusion test is not quite sufficient for producing the sensitivity outcome for adequate therapy. For the appropriate treatment of enteric fever, it is useful to determine the minimum inhibitory concentration of the antibiotics in order to curtail the inappropriate selection of the drug and to reduce the resistance rate that arises due to the rampant use of antimicrobial agents. Further analysis of risk factors, treatment outcome, and molecular epidemiology of* Salmonella enterica* with larger patient population will be highly effective in formulation of policies for the management of enteric fever cases in our country.

## Figures and Tables

**Table 1:** Antimicrobial susceptibility pattern of *Salmonella* isolates.

Antimicrobials	Susceptibility of *Salmonella* Typhi			Susceptibility of *Salmonella* Paratyphi		
	(*n* = 74)			(*n* = 14)		
	Sensitive	Intermediate	Resistant	Sensitive	Intermediate	Resistant
	*N* (%)	*N* (%)	*N* (%)	*N* (%)	*N* (%)	*N* (%)
Ampicillin	70 (94.6)	2 (2.7)	2 (2.7)	14 (100)	—	—
Azithromycin	74 (100)	—	—	14 (100)	—	—
Chloramphenicol	74 (100)	—	—	14 (100)	—	—
Cotrimoxazole	74 (100)	—	—	14 (100)	—	—
Cefixime	74 (100)	—	—	14 (100)	—	—
Ceftriaxone	74 (100)	—	—	14 (100)	—	—
Tetracycline	72 (97.3)	2 (2.7)	—	14 (100)	—	—
Levofloxacin	16 (21.6)	52 (70.3)	6 (8.1)	8 (57.1)	6 (42.9)	—
Ciprofloxacin	16 (21.6)	52 (70.3)	6 (8.1)	8 (57.1)	6 (42.9)	—
Nalidixic acid	16 (21.6)	—	58 (78.4)	8 (57.1)	—	6 (42.9)

**Table 2:** MIC distribution of *Salmonella* isolates.

Antibiotic (breakpoint)	Bacterial isolates	MIC (*µ*g/ml)										Total
		0.0075	0.015	0.03	0.06	0.125	0.5	1	2	4	8	
AZM (≤16 *µ*g/ml)	*S.* Typhi	—	—	—	—	37	19	17	1	—	—	74
	*S.* Paratyphi	—	—	—	—	7	—	2	5	—	—	14
CFM (≤1 *µ*g/ml)	*S.* Typhi	9	2	18	16	20	9	—	—	—	—	74
	*S.* Paratyphi	—	—	7	6	1	—	—	—	—	—	14
C (≤8 *µ*g/ml)	*S.* Typhi	—	—	—	—	—	—	—	18	29	27	74
	*S.* Paratyphi	—	—	—	—	—	—	—	—	5	9	14
CIP (≤0.06 *µ*g/ml)	*S.* Typhi	8	4	18	38	—	2	4	—	—	—	74
	*S.* Paratyphi	4	4	6	—	—	—	—	—	—	—	14

*S.*: *Salmonella*, AZM: azithromycin, CFM: cefixime, C: chloramphenicol, CIP: ciprofloxacin, and MIC: minimum inhibitory concentration.

**Table 3:** Indicators of *Salmonella* isolates with nalidixic acid and ciprofloxacin susceptibility.

Indicators	DDST	MIC (CIP only)
NA and CIP sensitive isolates	24 (27.3%)	24 (27.3%)
NA and CIP resistant isolates	6 (6.8%)	4 (4.5%)
NA resistant and CIP intermediate isolates	58 (65.9%)	2 (2.3%)
NA resistant and CIP sensitive isolates	—	58 (65.9%)

NA: nalidixic acid, CIP: ciprofloxacin, DDST: disk diffusion susceptibility testing, and MIC: minimum inhibitory concentration.

## Abbreviations

ASM: American Society for Microbiology
ATCC: American type culture collection
BHI: Brain heart infusion broth
CLSI: Clinical and Laboratory Standard Institute
FQs: Fluoroquinolones
MDR: Multidrug resistant
MIC: Minimum inhibitory concentration
NARS: Nalidixic acid resistant* Salmonella*
*S.* Typhi: * Salmonella enterica* subspecies enterica serovar Typhi
*S.* Paratyphi: * Salmonella enterica* subspecies enterica serovar Paratyphi
ZOI: Zone of inhibition.
